# The IL-17A-neutrophil axis promotes epithelial cell IL-33 production during nematode lung migration

**DOI:** 10.1016/j.mucimm.2023.09.006

**Published:** 2023-09-30

**Authors:** Jesuthas Ajendra, Pedro H. Papotto, James E. Parkinson, Rebecca J. Dodd, André L. Bombeiro, Stella Pearson, Brian H.K. Chan, Julie C. Ribot, Henry J. McSorley, Tara E. Sutherland, Judith E. Allen

**Affiliations:** 1 Lydia Becker Institute of Immunology and Inflammation, Wellcome Trust Centre of Cell Matrix Research, School of Biological Sciences, Faculty of Biology, Medicine and Health, University of Manchester, Manchester, United Kingdom; 2 Institute for Medical Microbiology, Immunology and Parasitology, University Hospital Bonn, Bonn, Germany; 3 Instituto de Medicina Molecular João Lobo Antunes, Faculdade de Medicina, Universidade de Lisboa, Av. Professor Egas Moniz, 1649-028 Lisboa, Portugal; 4 Division of Cell Signalling and Immunology, School of Life Sciences, University of Dundee, Dundee, United Kingdom; 5 School of Medicine, Medical Sciences and Dentistry, Institute of Medical Sciences, University of Aberdeen, Aberdeen, United Kingdom

## Abstract

The early migratory phase of pulmonary helminth infections is characterized by tissue injury leading to the release of the alarmin interleukin (IL)-33 and subsequent induction of type 2 immune responses. We recently described a role for IL-17A, through suppression of interferon (IFN)-γ, as an important inducer of type 2 responses during infection with the lung-migrating rodent nematode *Nippostrongylus brasiliensis*. Here, we aimed to investigate the interaction between IL-17A and IL-33 during the early lung migratory stages of *N. brasiliensis* infection. In this brief report, we demonstrate that deficiency of IL-17A leads to impaired IL-33 expression and secretion early in infection, independent of IL-17A suppression of IFN-γ. Neutrophil-depletion experiments, which dramatically reduce lung injury, revealed that neutrophils are primarily responsible for the IL-17A-dependent release of IL-33 into the airways. Taken together, our results reveal an IL-17A-neutrophil-axis that can drive IL-33 during helminth infection, highlighting an additional pathway by which IL-17A regulates pulmonary type 2 immunity.

## Introduction

Interleukin-33 (IL-33) belongs to the IL-1 cytokine family and plays a key role in innate and adaptive immunity. IL-33 signals via interleukin-1 receptor-like 1 (ST2), which is expressed on many different cell types including eosinophils, group 2 innate lymphoid cells (ILC2), and T helper (Th)2 cells^1^. The expression of ST2 on cell types is closely associated with type 2 immunity and evidence that IL-33 is a potent inducer of type 2 responses^2^ makes the IL-33/ST2 axis a therapeutic target in type 2 mediated diseases. However, IL-33 is implicated more broadly in the maintenance of tissue homeostasis and has roles in protection against microbial infection and regulatory T cell (Treg) expansion^3^. In contrast to most other cytokines, IL-33 is usually released during cellular necrosis, underpinning its role as an “alarmin” cytokine^4^. Studies with infection by the nematode *Strongyloides venezuelensis* or administration of the fungus *Alternaria alternata* have demonstrated IL-33 production specifically by cells of the airway epithelium including type 2 alveolar epithelial cells (AECs)^5,6^.

The pro-inflammatory cytokine IL-17A is typically associated with host protection against fungal and bacterial infections^7^. However, using a model of infection with the lung-migrating nematode *Nippostrongylus brasiliensis* (*Nb*), we recently demonstrated that IL-17A is also necessary to mount a pulmonary type 2 response^8,9^. In this helminth setting, IL-17A from innate γδ T cells exerts a suppressive effect on interferon (IFN)-γ released by multiple cell types early during infection^8^. This inhibition of IFN-γ allows development of the adaptive pulmonary Th2 response. However, existing data on the interaction between IL-17A and IL-33^10–13^ suggests the possibility that innate IL-17A during *Nb* infection may function beyond its ability to suppress IFN-γ. In a neonatal mouse model of influenza, infection-induced IL-17A was associated with increased IL-33 production by lung epithelial cells, subsequently generating a local type 2 immune response^10^. Similarly, mice lacking IL-17A exhibit decreased IL-33 in visceral adipose tissue, leading to reduced Treg expansion and failure to regulate thermogenesis^11^. In both these studies, as in lung *Nb* infection, the source of IL-17A is γδ T cells^7,8^. IL-17A-induced IL-33 also promotes type 2 immunity during atopic dermatitis with the IL-17A source being ILC3s^12^. Of note, during pulmonary *Aspergillus fumigatus* infection, IL-33 negatively regulates IL-17A production^13^, demonstrating cross-regulation between these two cytokines. During *Nb* infection, we have shown IL-17A as a driver of type 2 immunity^8,9^, but Hung et al.^14^ have demonstrated a pivotal role for IL-33 in the type 2 response in *Nb* infection. Given the existing literature on the interactions between IL-17A and IL-33 described above, we felt it essential to investigate the relationship between these two cytokines during the lung-migrating phase of *Nb* infection.

Here, we describe IL-17A-dependent IL-33 release by the lung epithelium during the early phase of *Nb* infection. However, this effect of IL-17A was independent of the IFN-γ-suppressing function. Consistent with this, IL-17A-induced neutrophilia, which is primarily responsible for lung damage in this model, was a key driver for IL-33 release but was not sufficient to drive the type 2 response. Together, our data demonstrate that IL-17A acts as an upstream regulator of type 2 immune responses in the lung through two distinct pathways: IFN-γ-suppression and IL-33 secretion.

## Material and Methods

### Mice and ethics

C57BL/6 J^crl^ mice were obtained from Charles River. B6.Il17a^tm1.1(icre)Stck^; B6.129Gt(ROSA)26Sor^tm1(EYFP)Cos^ mice were originally provided by Dr Brigitta Stockinger^15^. B6.Il17a^tm1.1(icre)Stck^; B6.129Gt(ROSA)26Sor^tm1(EYFP)Cos^ homozygote mice are IL-17A-deficient and described here as *Il17a*-KO. Rag1-OTII (B6.129S7-^Rag1tm1Mom^ Tg(TcraTcrb)425Cbn), described here as RAG-KO, were kindly provided by Dr Jo Konkel and Dr John Grainger. C57BL/6-*Il17a*^*tm1Bcgen*^/J (*Il17a-*GFP reporters) were originally made by Biocytogen. Male and female mice were age- and sex-matched and housed in individually ventilated cages. Experimental mice were not randomized in cages, but each cage was randomly assigned to a treatment group. Mice were culled by asphyxiation in a rising concentration of CO_2_. Experiments were performed in accordance with the United Kingdom Animals (Scientific Procedures) Act of 1986 (project license number 70/8547 and PP4115856) or were approved by the institutional animal welfare body (ORBEA-iMM) and by the DGAV (Portuguese competent authority for animal protection), in accordance with Directive 2010/63/EU.

#### *N. brasiliensis* infection

*Nb* was maintained by serial passage through Sprague-Dawley rats, as described^16^. Third-stage larvae (L3) were isolated from the clean edges of parasite cultures and washed five times with phosphate buffered saline (PBS, Dulbecco’s PBS, Sigma) before injection. On day 0, mice were injected subcutaneously with 250 larvae (L3) in the scruff of the neck. At various time points, mice were euthanized and lungs were taken for further analysis.

### Flow cytometry

Single-cell suspensions of the lung were prepared and stained for flow cytometry as previously described^8^ (Antibodies in Supplementary Table 1). For intracellular staining of IL-33 (clone: 396118, Invitrogen) by epithelial cell adhesion molecule (EpCAM) positive cells, a previously described protocol was used.^17^ For detailed stromal cell analysis, lungs were collected in PBS and then weighed and minced with blunt nose scissors. Tissue was then digested with Collagenase A (250 U/mL, Roche Diagnostics, 10103578001), Dispase (6 U/mL Dispase II, 04942078001, Roche Diagnostics), and DNAse I (250 U/mL DnaseI, Invitrogen 18047019) for 60 minutes, pipetting up and down every 20 minutes to agitate. Tissues were then mashed through a 70 μm cell strainer and red blood cells were lysed with ammonium-chloride-potassium (ACK) lysis buffer in a protocol modified from^18,19^. For cytokine analysis cells were stimulated for 4 hours at 37 °C with cell stimulation cocktail containing protein transport inhibitor (eBioscience), then stained with live/dead (Thermo Fisher Scientific). After surface antibody staining, cells were fixed o/n at 4 °C using ICC Fixation buffer (eBio-science), then incubated for 20 minutes at room temperature (RT) in permeabilization buffer (eBioscience). Intracellular staining was performed for IL-33 for 30 minutes at RT. Samples were acquired with an LSR Fortessa II flow cytometer and data was analyzed using FlowJo V9 software (Treestar).

### Histology, immunofluorescence, and lacunarity measurement

For histology, the left lung lobe was inflated with PBS and fixed in formalin for 24 hours before transferring to 70% ethanol. Tissues were processed, embedded in paraffin, and then sectioned (5 μm thickness) onto glass microscopy slides (Superfrost Plus Adhesion slides, J1830AMNZ, Thermo Fisher Scientific). For immunostaining, lung sections were deparaffinized, and rehydrated, and heat-mediated antigen retrieval performed using 10 mM sodium citrate buffer, pH 6.0 (20 minutes incubation, 95 °C). Tissue sections were permeabilized in 0.5% Triton-X100in PBS (20 minutes, RT) and then non-specific protein-binding blocked (10% normal donkey serum, 1%BSA, 0.05% Tween-20 in PBS, 1 hour at RT) before sections were incubated with primary antibody (Supplementary Table 2) overnight at 4 °C. All sections were washed in PBS with 0.05% Tween-20 followed by PBS before incubation with secondary antibody (donkey-anti-rabbit Northern Lights NL637-conjugated antibody, 1:200, NL005; R&D Systems, or streptavidin NL557-conjugated, 1:800, NL999, R&D Systems) for 1 hour at RT. Sections were washed as before, incubated with 4′,6-diamidino-2-phenylindole (DAPI, 1:40,000 in PBS, 2 minutes, RT), and then mounted (Fluorescence mounting medium, S3023, Agilent Technologies), and cover-slipped. Staining was visualized on an EVOS FL Imaging System (Thermo Fisher Scientific) and image analysis was performed using FIJI software (Version 1.54f 29). For bronchial epithelial regions, regions of interest (ROI) were drawn around the airways, and the integrated density was calculated. This was then normalized to the total length of the airway basement membrane to give a normalized integrated density per mm. For parenchyma, type 2 AEC staining nuclei were segmented and isolated, and then an integrated density was calculated for each cell nuclei. These were then averaged across samples and plotted as raw integrated densities.

For all these images background was subtracted based on negative controls and images were brightened to a minimum threshold. IL-33 KO mice, kindly provided by Dr. John Grainger (Lydia Becker Institute, Manchester, UK) were used to validate specificity of IL-33 staining protocols.

### IL-17A reporter staining

For IL-17A+TCR γδ^+^ staining, *Il17a*-GFP reporter mice were used. Day 1 (d1) post-infection (pi) mice were euthanized by CO_2_ inhalation and lungs were perfused with fixative solution (PFA 4% in PBS 0.1M). Lungs were post-fixed in PFA 4% (o/n, 4 °C), rinsed with sucrose 30% (w/v, diluted in PBS 0.1M; 72 hours, 4 °C) and frozen in OCT compound (−37 °C). Samples were longitudinally cut (12 μm slides), blocked (BSA 3% in PBS 0.1 M plus anti-mouse clusters of differentiation (CD)16/CD32 1:100; 45 minutes, RT) and incubated with the following antibodies (diluted in PBS 0.1M containing BSA 1% and Triton-X 0.02%; over-night, 4 °C): PE anti-mouse TCR-gamma/delta (Invitrogen, Ref 12-5711-82, clone GL3; 1:100) and Alexa Fluor 488 anti-GFP (Invitrogen, Ref A21311; 1:200). After washes, coverslips were mounted with glycerol solution (containing DAPI 1:1000). Samples were analyzed in a widefield fluorescence imaging system (Zeiss Cell Discoverer 7). Antibody specificity was previously checked by flow cytometry and by their binding to negative control tissues from TCRγδ^−^/GFP^−^ mice. Fluorescence control samples were prepared by primary antibody omission.

### Lung damage analysis

For measurement of lacunarity, paraffin lung sections were stained with hematoxylin and eosin using standard protocols. Sections were imaged using a 3DHistech Pannoramic P250 slide scanner and images were processed in a KNIME software work-flow to obtain 50 random ROIs across the whole lung section. ROIs that contained lobe boundaries or extensive artifacts were excluded from the analysis. The ROIs were then converted to binary images and lacunarity was quantified using the FracLac plugin for ImageJ^20^. The values of all the ROIs were averaged to obtain estimates for the entire lobe.

### Neutrophil depletion

Neutrophil depletion was performed as described before^8^. In short, mice were injected intraperitoneally with either 250 mg or 500 mg of neutrophil-depleting antibody (InVivoMAb clone 1A8, BioXcell) days −1 and 1 pi. Control mice were treated with corresponding isotype control (InVivoMAb rat IgG2a isotype control, anti-trinitrophenol, BioXcell).

### IL-33 inhibition

IL-33 was suppressed using *Heligmosomoides polygyrus* alarmin release inhibitor (HpARI), which was generated as previously described^21^. HpARI (10 μg) was administered intranasally, after brief anesthesia with Isoflurane, in 30 μl of PBS on d0 and d1 pi with *Nb*. Controls were treated with 30 μl PBS intranasally.

### IL-33 ELISA

Bronchoalveolar lavage (BAL) was collected using PBS via cannulation of the trachea. Collected BAL fluid was centrifuged at 400 xg for 5 minutes to pellet the cells, and supernatants were collected and frozen at −80 °C until required. IL-33 concentrations were measured by ELISA (IL-33 Duoset, R&D Systems) and detected using horseradish peroxidase-conjugated streptavidin and 3,3′,5,5′-Tetramethylbenzidine (TMB) substrate (BioLegend) and the reaction stopped with 0.18M H_2_SO_4_. Absorbance was measured at 450 nm using a VersaMax microplate reader (Molecular Devices) with a background measurement at 570 nm. Data was calculated as the 450 nm value–570 nm value and then the optical density (OD) of a PBS blank well was subtracted from each sample. Five-point curves were generated, and unknown values interpolated from this curve.

### Statistics

Prism 9.5.1 (version 9, GraphPad Software Inc.) was used for statistical analysis. Data were tested for normality using the Shapiro-Wilk test and then differences between experimental groups were assessed by either one-way analysis of variance with Tukey’s multiple comparisons for parametric data or Kruskal-Wallis test with Dunn’s multiple comparisons for non-parametric data. For gene expression data, values were log2 transformed to achieve normal distribution. Comparisons with a value < 0.05 were considered to be statistically significant. Data are represented as mean ± SD.

## Results

### Lack of IL-17A leads to impaired early IL-33 production during *Nb* infection

**Fig. 1:**
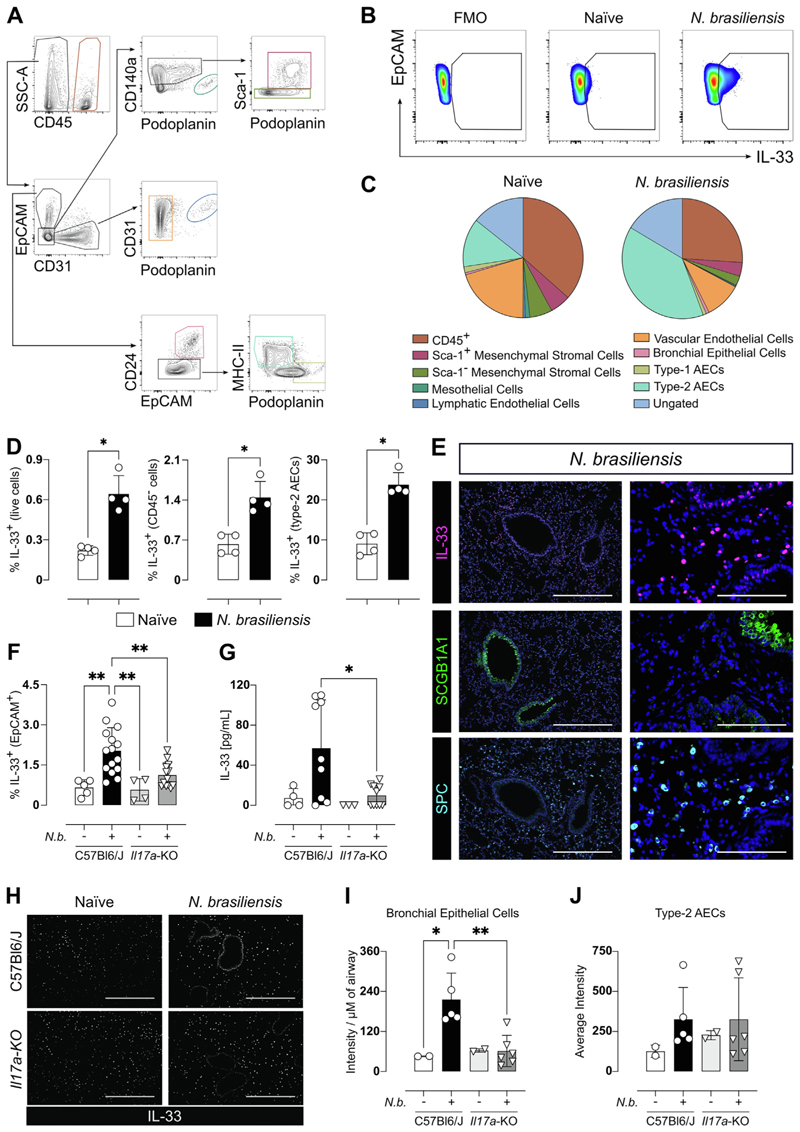
Type 2 AECs are the main IL-33 producers during the lung phase of *Nb* infection and IL-I7A-deficiency reduces IL-33 production. Mice were infected with 250 L3 *Nb* and lungs analyzed for IL-33 expression at day 1 post-infection. Representative flow cytometry gating strategy for CD45-lung stromal cell populations (A). Representative plots showing frequency of IL-33^+^ cells within the EpCAM+ population in naïve and *Nb-*infected animals (B). Pie charts showing the relative contribution of cell populations to total IL-33 expression in the lungs of naïve and *Nb-*infected mice (C). Frequency of IL-33+ cells within live cells, CD45-cells, and type 2 AECs in naïve and *Nb-*infected WT mice (D). Representative histological IF images of FFPE lung sections stained with IL-33 (magenta), surfactant protein C (SPC, cyan), Scgb1a1 (green) and DAPI (blue) at two different magnifications (E) scale bars are 400 μm and 100 μm for left and right images respectively. WT and *Il17a*-KO mice were infected with 250 *Nb* L3 larvae and IL-33 production on day 2 was assessed. Frequency of IL-33 in Epcam^+^CD45^-^ lung epithelial cells in WT and *Il17a*-KO (F) and IL-33 in the BAL fluid of naïve and mice 1 day post-infection (G). Microscopy images of IF staining for IL-33 (white) in lung sections of WT and *Il17a*-KO mice 1 day post *Nb* infection and naïve controls. <scale bars: 400 μm> (H). Quantification for IL-33 positive staining in the bronchial epithelium (I) and nuclei of type 2 AECs (J). Data in D are representative of three individual experiments. Data in C and F are pooled from three individual experiments. Data in G, I, and J are pooled from 2 independent experiments. Data are expressed as mean ± SD and were tested for normality and then analyzed by Kruskal-Wallis with Dunn’s multiple comparison test or one-way analysis of variance as appropriate. *P < 0.05, **P < 0.01. Antibody-positive staining area was normalized by the length of the airway for the bronchial epithelium and calculated per average of the segmented nuclei for the parenchyma.

Stromal and epithelial cells are important sources of IL-33 during infection with *Nb*^22^. To better understand the specific cellular sources of IL-33 early following infection, we used a flow cytometric approach to assess the CD45^−^ (non-immune) population (Fig. 1A and B)^19^. In the naïve state, most IL-33 positive cells included both type-1 (CD45^-^EpCAM^+^PDPN^+^) and type-2 AECs (CD45^-^EpCAM^+^MHCII^+^), along with vascular endothelial cells (CD45^−^CD31^+^EpCAM^−^). Additionally, various other compartments of the lung epithelium, such as bronchial epithelial cells (CD45^−^EpCAM^+^CD24^+^), mesothelial cells, (CD45^−^EpCAM^−^CD31^−^-PDPN^+^), as well as Sca-1^+^ and Sca-1^−^ mesenchymal stromal cells (CD45^−^EpCAM^−^CD31^−^CD140a^+^), were found to express IL-33, as illustrated in Fig. 1C. At 1 day after *Nb* infection, the proportion of cells that expressed IL-33 significantly increased in the CD45−population while decreasing in the CD45+ population (Fig. 1C and D). Type-2 AECs exhibited the largest increase in IL-33 expression relative to naïve mice and became the dominant IL-33-expressing cell type in infected mice (Fig. 1C and D), consistent with studies on infection with the nematode *S. venezuelensis*^5^.

To further localize IL-33 expression, we performed immunofluorescence (IF) staining on sections obtained from *Nb-*infected mice on day 2 (d2) pi. Sequential sections were stained for surfactant-protein C (SPC), which is selectively expressed by type 2 AECs^23^ in the lung parenchyma and Scg-b1a1, which identifies club cells of the bronchial epithelium lining the airways^24^. A side-by-side comparison with IL-33 staining revealed co-localization of IL-33 with both SPC and Scgb1a1 (Fig. 1E), supporting our flow cytometry data. Recently, adventitial fibroblasts were shown to contribute to IL-33 production in the lung^25^. However, while we could observe overlapping of S100a4^+^ cells and IL-33 (Supplementary Fig. 1A), this was a fairly rare population in comparison type 2 AECs and was consistent with the small population of IL-33^+^ PDGFRα^+^ cells identified by flow cytometry. Although we focused on non-immune cells, previous data suggested the possibility that myeloid cells could be a key source of IL-33 expression^17,26^ within the lung parenchyma. At d2 post *Nb* infection, most myeloid cells in the lung are alveolar macrophages and neutrophils, which both constitutively express Ym1^27^. We therefore co-stained lung sections for both Ym1 (pink) and IL-33 (yellow), which revealed no co-staining, (Supplementary Fig. 1B) suggesting that the source of IL-33 in the parenchyma of *Nb-*infected mice was not myeloid cells.

We recently described IL-17A as an important initiator of type 2 responses in the lung by downregulating early IFN-γ production^8^. To investigate the relationship between IL-17A and IL-33 during the early phase of infection, both *Il17a*-KO and wild-type (WT) mice were infected with 250 L3 *Nb* larvae and the pulmonary immune response was assessed at d1 and d2 pi. Using flow cytometry, we found that the infection-driven IL-33 produced by EpCAM^+^CD45^−^ cells was significantly reduced in *Il17a*-KO mice compared to WT controls (Fig. 1F). The difference in the frequency of IL-33^+^ EpCAM^+^CD45^−^ cells between WT and *Il17a*-KO mice was observed as early as 16 hours, possibly correlating with the time point *Nb* larvae enter the lung (Supplementary Fig. 1C). Using ELISA, we determined that by 24 hours pi WT mice secreted significantly higher amounts of IL-33 into the BAL fluid compared to infected *Il17a*-KO mice (Fig. 1G). Type 2 AECs are likely to be the most abundant source of secreted IL-33 based both on our flow cytometry data (Fig. 1) and literature reports^5,28^. However, by IF staining of lung sections, reduced IL-33 expression in *Il17a*-KO relative to WT mice was evident only in the bronchial epithelial cells of the airways (Fig. 1H and I) and not in the parenchymal AECs (Fig. 1H and J). This may be due to the reduced dynamic range of IF staining relative to flow cytometry and/or differences in tissue processing between these methods. However, bronchial epithelial cells represent less than 1% of IL-33^+^ cells by flow cytometry and as such cannot fully explain the reduction in the frequency of IL-33^+^ EpCAM^+^CD45^−^ cells between WT and *Il17a*-KO mice. Taken together, these data reveal an IL-33 regulating function of IL-17A during the early stages of *Nb* infection.

**Fig. 2:**
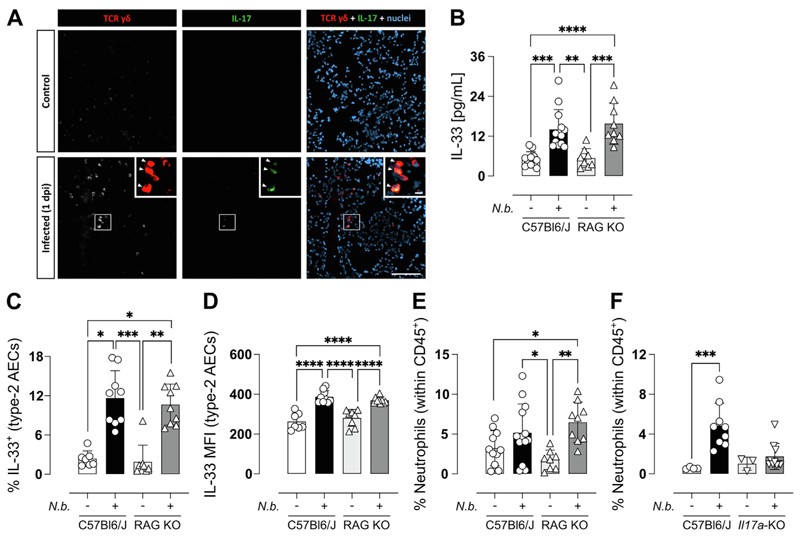
Lung γδ T cells induce IL-17A upon *Nb* infection, which recruits neutrophils, driving tissue damage and release of IL-33 into the BAL. IF of lung γδ T cells (TCRγδ, red) in the lungs of naïve and infected *Il17a-*GFP mice. Arrowheads point at IL-17^+^ γδ T cells. <scale bars: 100 μm; inset, 10 μm> (A). IL-33 in the BAL fluid of naïve and infected WT and RAG-KO mice day 1 post-infection quantified by ELISA (B). WT and RAG-KO mice were infected with 250 *Nb* L3 larvae and immune responses were measured on day 1 post-infection. Frequency (C) and mean fluorescence intensity (D) of IL-33^+^ in type 2 AECs and frequency of neutrophils in the BAL of RAG-KO (D) and *Il17a*-KO mice (F) during *Nb* infection. Data in B-F is pooled from two separate experiments. Data are expressed as mean ± SD and are representative of 2 individual experiments and were tested for normality and then analyzed by Kruskal-Wallis with Dunn’s multiple comparison test or one-way analysis of variance as appropriate. *P < 0.05, **P < 0.01.

We previously identified γδ T cells as the key source of IL-17A during *Nb* infection^9^. To determine whether IL-17A^+^ γδ T cells were localized around areas in the lung where IL-33 expression was observed, we stained for γδ T cells within the lung tissue of both infected and non-infected (control) mice (Fig. 2A). The immunostaining signal for TCR γδ was more intense in the infected group and using eGFP IL-17 reporters, IL-17-producing γδ T cells were only detected in the infected lungs and were absent in the control tissue (Fig 2A). γδ T cells were observed in the damaged parenchyma but were not in the alveoli lumen. Autofluorescence emitted by erythrocytes and mucus precluded an assessment of γδ T cells in blood vessels or damaged airways.

To validate the role of IL-17A-producing γδ T cells in the regulation of IL-33 we chose to first examine RAG-KO mice, which lack B and T cell populations including γδ T cells but do have other innate IL-17-producing cells. Upon examining the lungs of *Nb-*infected RAG-KO mice, we did not observe a reduction in IL-33 release into the BAL (Fig. 2B). In addition, both RAG-KO and WT mice exhibited an increase in the percentage and mean fluorescence intensity of IL-33 expression within type 2 AECs following *Nb* infection (Fig. 2C and D). Notably, no differences were observed in IL-33 expression between infected WT and infected RAG-KO mice, with similar levels of IL-33 expression observed in both groups. While the absence of IL-17A-producing γδ T cells in RAG-KO mice did not significantly impact IL-33 expression during *Nb* infection, it was possible that compensatory IL-17 mechanisms were at play as previously shown^29^. During the early phases of *Nb* infection, neutrophils are rapidly recruited to the lungs, with peak-neutrophilia being between d1 and d2 pi^30^, a response that requires IL-17 receptor ^31^. Nonetheless, despite the lack of γδ T cells, following *Nb* infection, RAG-KO mice still displayed increased neutrophils recruitment (Fig. 2E), in direct contrast to the lack of neutrophils in *Il17a*-KO mice (Fig. 2F)^9^. Neutrophils primed by *Nb* infection have been shown to upregulate *Il33* transcripts^32^ and are a major driver of tissue injury which in turn could lead to IL-33 release. Therefore, we considered that the limited neutrophil recruitment in *Il17a*-KO mice during *Nb* infection might explain their failure to increase IL-33 levels in the lung.

### IL-17A-dependent neutrophilia is a driver of IL-33 release into the BAL

**Fig. 3:**
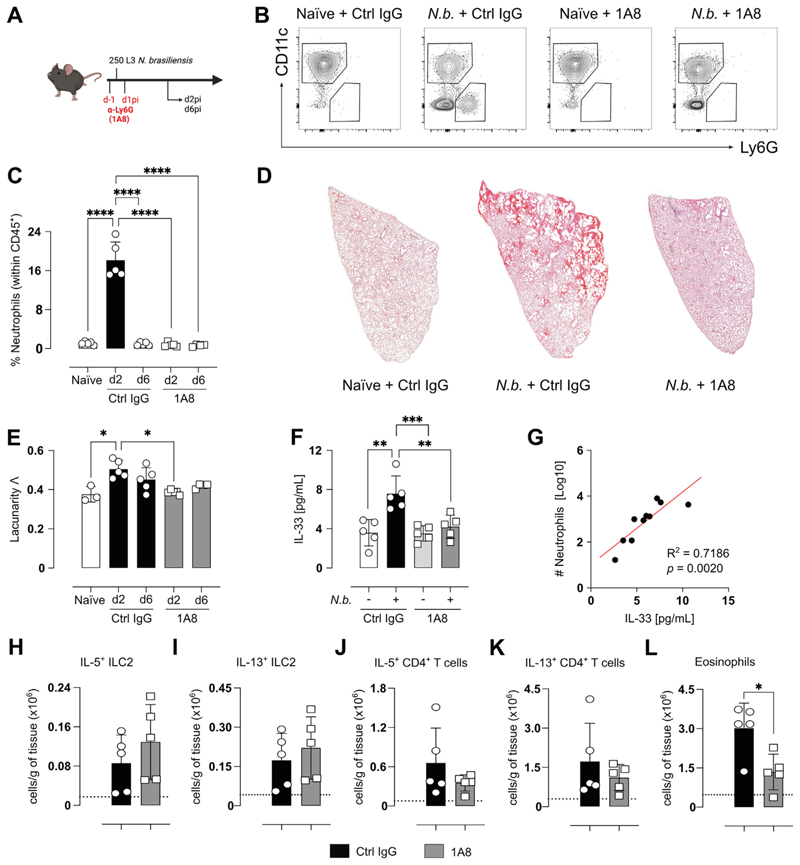
Neutrophil depletion reduces tissue damage but does not inhibit the development of the Th2 response. C57BL/6J mice were infected with 250 *Nb* L3 larvae and mice were injected intraperitoneally with a-Ly6G (1A8) or corresponding isotype control 1 day before and 1 day after infection then analyzed at day 2 or day 6, Schematic representation of dosing and infection strategy alongside analysis timepoints (A). Confirmation of neutrophil (Ly6G^+^) accumulation and antibody-dependent depletion via representative flow cytometry plots (B) and quantification of neutrophil frequency in the BAL (C). Representative images of lung sections stained with hematoxylin & eosin at day 2 post-infection (D) and quantification of lacunarity (L) on day 2 and 6 post-infection (E). IL-33 in the BAL fluid at day 2 quantified via ELISA (L) and correlation between these BAL IL-33 levels and neutrophil cell counts (G). Numbers of IL-5^+^ (H) and IL-13^+^ (I) ILC2, as well as IL-5^+^ (J) and IL-13^+^ (K) CD4^+^T cells at day 6 alongside lung tissue eosinophils (E). Dotted lines represent the mean of the WT naïve control group. Data in B is representative of two experiments. Data in F and G are from one experiment. Data in C, D, and E are representative of two experiments. H, I, J, and K data are representative of two experiments. Data are expressed as mean ± SD and were tested for normality and then analyzed by Kruskal-Wallis with Dunn’s multiple comparison test or one-way analysis of variance as appropriate. *P < 0.05, **P < 0.01, ***P < 0.001, ****P < 0.0001.

To investigate whether neutrophils were responsible for enhanced IL-33 levels during infection, neutrophils were depleted at d-1 and d1 pi (Fig. 3A). Injection of anti-Ly6G effectively prevented neutrophil accumulation in the BAL at d2pi compared to isotype control (Fig. 3B and C). Histological sections of the lungs demonstrated infection-induced injury and hemorrhage at d2pi in isotype-treated WT mice, an effect that is almost absent in infected mice depleted of neutrophils (Fig. 3D). Using measures of lacunarity as an indicator of acute lung injury^20^, neutrophil-depleted mice displayed no significant signs of damage (Fig. 3E). These findings reinforce previous observations that neutrophilia is largely responsible for the severe lung damage observed in this model^31^. Consistent with the decreased tissue damage upon neutrophil depletion, we observed a significant alteration in the levels of secreted IL-33 in the BAL on d2pi. Mice that were depleted of neutrophils exhibited a substantial reduction in IL-33 secretion into the BAL fluid compared to the isotype controls (Fig. 3F). The specific role of neutrophils in regulating IL-33 levels was supported by a significant positive correlation between the number of neutrophils in the BAL and IL-33 concentration (Fig. 3G). Mice depleted of neutrophils displayed less tissue damage during *Nb* infection, which consequently led to significantly lower levels of IL-33 protein in the BAL fluid. This compelling evidence suggests that neutrophil-mediated tissue damage serves as a critical inducer of IL-33 release. Of note, increased bleeding into the BAL has been observed in IL-33 deficient mice at d3 after *Nb* infection^14^, suggesting that the IL-33 induced by neutrophilia is involved in limiting the early damage neutrophils cause, consistent with pro-reparative functions of IL-33^2^. However, despite clear differences in IL-33 secreted into the BAL, no significant difference was observed between the treatment groups in lung sections stained for IL-33 (Supplementary Fig. 1D and E).

The differences we have observed in IL-33 expression in different epithelial cells may relate to very fine differences in timing of experiments. The larvae will enter the lung parenchyma first before breaking their way into the epithelium, so d1 or d2 may be too late to see the effect of worms on IL-33 expressing type 2 AECs because the parasites have already moved toward the bronchial airways. Hung et al. have demonstrated that the context and cell source of IL-33 can influence its function in anti-helminth immunity^26^. Thus, understanding the specific consequences of both epithelial cell-derived and damage-derived IL-33 is important. While our data strongly implicates neutrophil-mediated cellular damage as the major IL-17-dependent driver of IL-33 release, changes in epithelial cell expression suggest other potential pathways. For example, IL-17A may create an environment more permissive for IL-33 production, as suggested by work on human epidermal keratinocytes in which IL-17A causes phosphorylation of EGFR, ERK, p38 and STAT1, necessary for the induction of IL-33^33^.

Since the absence of neutrophils led to decreased IL-33 secretion, we next examined the effect of the neutrophil depletion on the subsequent type 2 response, which peaks around 6 days post *Nb* infection. Despite the reduced IL-33 levels, no differences were observed in important type 2 parameters such as IL-5^+^ ILC2 (Fig. 3H), IL-13^+^ ILC2 (Fig. 3I), IL-5^+^CD4^+^ T cells (Fig. 3J) and IL-13^+^CD4^+^ T cells (Fig. 3K). One exception was eosinophil numbers, which although increased in both groups over uninfected mice, were significantly lower in neutrophil-depleted mice compared to isotype-treated group (Fig. 3L).

### Suppressing IL-33 during *Nb* infection does not increase IFN-γ production

**Fig. 4:**
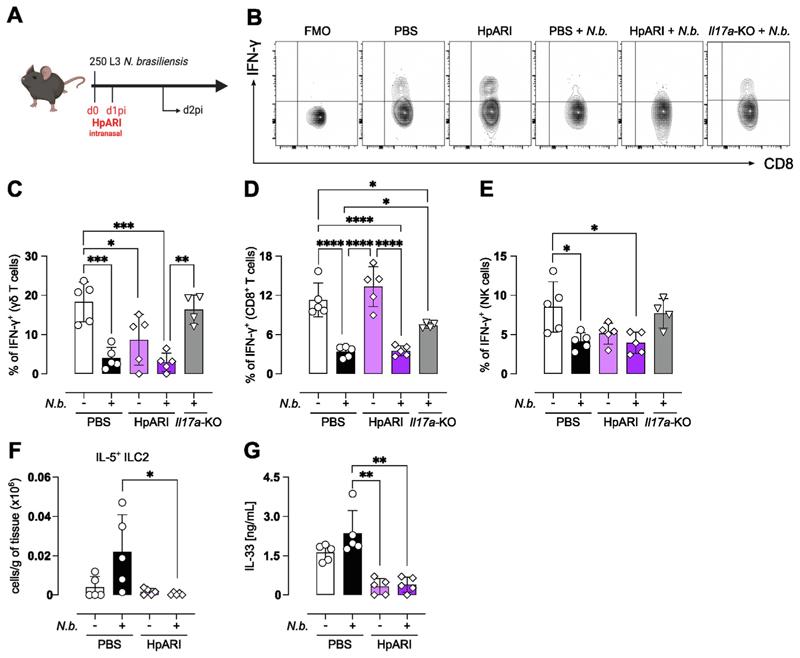
Blockade of IL-33 does not suppress IFN-γ and cannot prevent the development of a Th2 response. Mice were infected with 250 L3 *Nb* larvae and tissue analyzed on day 2 post-infection. Schematic showing the treatment regime of blocking with HpARI (A). Mice were infected with 250 *Nb* L3 larvae and either treated with intranasal HpARI or PBS on the day of and 1 day after, infection. Cellular sources of IFN-γ were analyzed via flow cytometry. Representative flow plots of IFN-γ production by single live TCRβ^+^CD8^+^ T cells alongside a fluorescence minus one (FMO) control (B). Quantification of the frequency of intracellular IFN-γ by γδ T cells (C), CD8^+^ T cells (D), and NK cells (E). *Il17a*-KO mice were included as controls because of their published failure to suppress IFN-γ in this model. Absolute count of IL5^+^ILC2 in the lung of naïve and day 2 *Nb-*infected mice (F) and IL-33 in the BAL fluid of naïve and day 2 post-infection animals as quantified via ELISA (G). Data in B, C, D, E, F, and G are representative of three experiments. Data are expressed as mean ± SD and were tested for normality and then analyzed by Kruskal-Wallis with Dunn’s multiple comparison test or one-way analysis of variance as appropriate. *P < 0.05, **P < 0.01, ***P < 0.001, ****P < 0.0001.

The neutrophil data led us to ask why the decrease in IL-33 after neutrophil depletion did not result in a subsequently impaired type 2 response. We have reported that during *Nb* infection, early IL-17A is responsible for the suppression of IFN-γ from all cellular sources^8^. Here we have observed that in the absence of IL-17A, IL-33 expression in the lung epithelium and IL-33 secretion into the BAL fluid is reduced (Fig. 1F–I). We therefore assessed the possibility that the ability of IL-17A to limit IFN-γ production may be acting indirectly through promotion of IL-33. To answer this question, we used a known suppressor of IL-33, the *H. polygyrus*-derived protein HpARI. HpARI directly binds to IL-33 and nuclear DNA, blocking the interaction of IL-33 with the ST2 receptor^21^. Furthermore, HpARI prevents the release of IL-33 from necrotic cells^21^. HpARI was administered intranasally at day 0 and day 1 of *Nb* infection and IFN-γ production was investigated at d2pi (Fig. 4A). Consistent with our previous findings^8^, at d2pi there was a decrease in the proportion of CD8^+^ T cells, γδ T cells, and NK cells producing IFN-γ (Fig. 4B-E).

Notably, blocking IL-33 with HpARI in *Nb*-infected mice did not reverse the suppression of IFN-γ (Fig. 4B-E). In contrast and as expected, IFN-γ production was increased in *Il17a*-KO mice compared to WT controls at d2pi with *Nb*. To validate that HpARI treatment was successfully blocking IL-33, we analyzed the numbers of ILC2s in the lung and observed a significant reduction in IL-5^+^ ILC2s in the lung at d2pi (Fig. 4F). Additionally, detectable IL-33 protein was significantly reduced in lung homogenates after HpARI treatment (Fig. 4G). Of note, existing data on the relationship between IL-33 and IFN-γ suggests that IL-33 induces IFN-γ from T cells and NK cells at least in the context of viral infection^34,35^. Thus, our finding that blocking IL-33 does not increase IFN-γ is consistent with this literature that IL-33 is a driver not an inhibitor of IFN-γ^34,35^.

The lung epithelium is a major barrier surface and is known to respond to IL-17A by producing antimicrobial proteins or neutrophil chemoattractants^36^. Our current data, along with other recent studies^10^ suggest that IL-17A regulation of the lung epithelium extends beyond the induction of pro-inflammatory and antimicrobial factors and involves regulation of type 2-associated molecules. Previously we demonstrated that IL-17A promotes pulmonary type 2 immune responses during *Nb* infection by downregulating early IFN-γ^8^, while here we show that IL-17A promotes IL-33 production mainly through neutrophil-mediated damage in response to incoming larvae. Because IL-33 can itself induce neutrophilia, IL-17A may be initiating a positive feedback loop^37,38^. The IL-33 suppressor HpARI did not result in changes in IFN-γ levels suggesting that IL-17A-dependent IL-33 production is independent from IL-I7A-dependent IFN-γ suppression. In addition, blocking IFN-γ rescues type 2 immunity in *Il17a*-KO mice^8^, indicating that IL-17A-mediated suppression of IFN-γ is sufficient to initiate type 2 responses without needing to enhance IL-33 expression. This is supported by our finding that the significantly reduced IL-33 in neutrophil-depleted mice did not prevent the induction of type 2 immune response to *Nb* infection. However, there is excellent evidence from gene-deficient mice for the importance of IL-33 in driving type 2 immunity^22^ and neutrophil depletion does not ablate IL-33. In addition, neutrophil-driven IL-33 release would be limited to the early damage at d1 and d2 pi. Once the adaptive immune response is established at d6, the impact of an early IL-33 burst may be less relevant. In line with the idea that timing is critical, work from Hung et al.^14^ shows that IL-33 selectively drives IL-13 production by ILC2 at d3, but does not prevent canonical Th2 responses at d6. In conclusion, our data supports a model in which IL-17 promotes type 2 immunity in the lung through early release of neutrophil-driven IL-33 but that suppression of IFN-γ is needed to promote the full Th2 response. However, much remains to be explored in the context of IL-17A-mediated induction of type 2 immunity including how and where these pathways overlap.

## Supplementary Material

Appendix
