# Supplementary material for: The IL-17A-neutrophil axis promotes epithelial cell IL-33 production during nematode lung migration
Source: Mucosal Immunol. Author manuscript; Available in PMC 2024 Aug 5. (PMC7616139; doi:10.1016/j.mucimm.2023.09.006)
Supplement: Appendix [file EMS196867-supplement-Appendix.pdf]

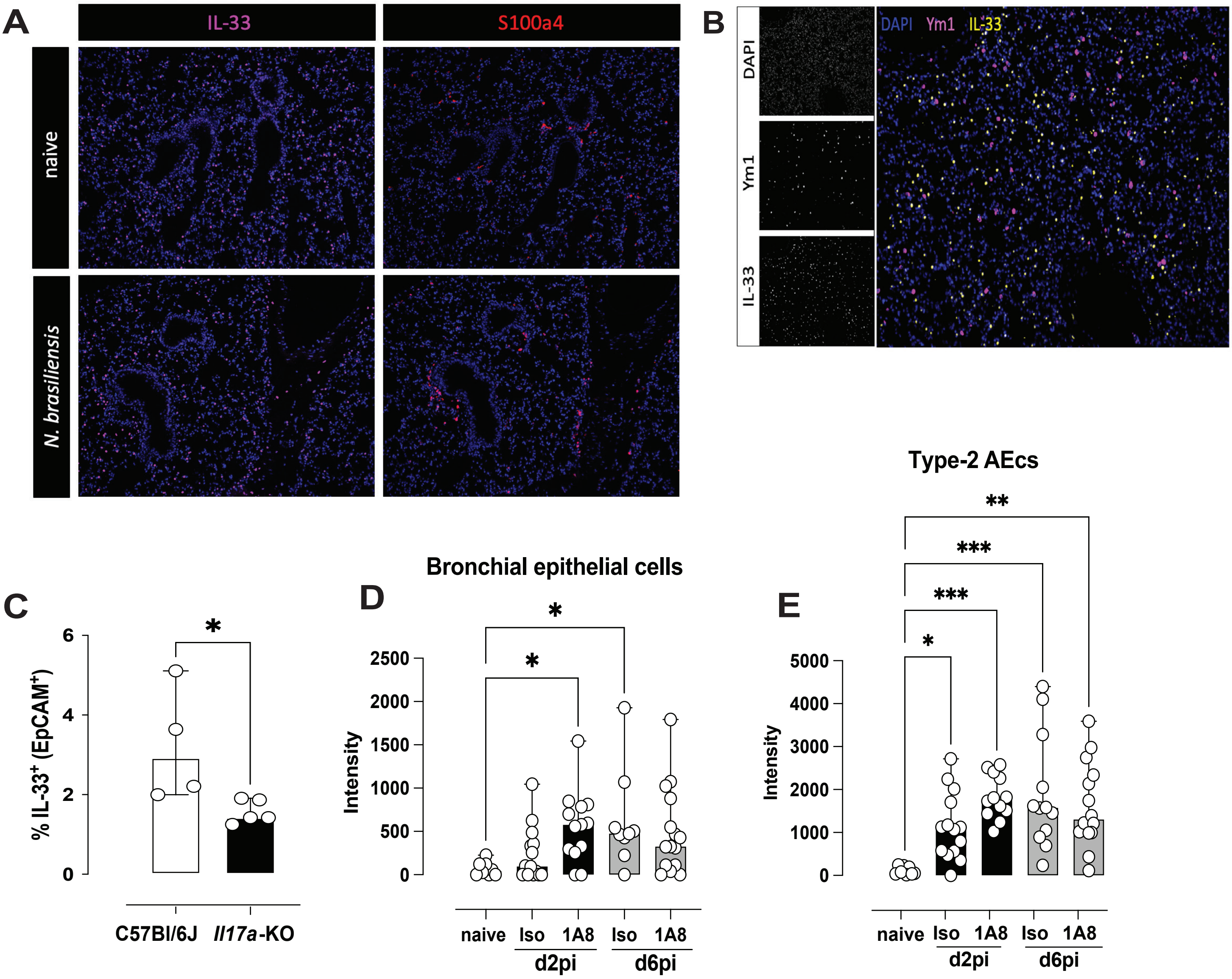

Supplementary Figure 1

IF images of FFPE lung sections of naive and *Nb* infected mice stained for IL-33 (magenta) and S100a4 (red) as well as DAPI (blue) (A). Co-staining of IL-33 (yellow) and Ym1 (pink) in *Nb* infected FFPE lung sections (B). IL-33 quantified from flow cytometry of epithelial cells of WT and *Il17a*-KO mice at 16 hrs post infecction with *Nb* (C). Quantification of IL-33 positive areas for bronchial epithelium (D) and type 2 AECs (E) for d2 and d6pi *Nb*, in naive mice an infected mice after neutrophil depletion (1A8) or isotype control treatment (Iso). Antibody positive staining area was normalized by the length the airway for the bronchial epithelium and calculated per average of the segmented nuclei for the parenchyma. Data are expressed as mean ± s.e.m. and is pooled from 2 independent experiments and were analysed by Kruskal-Wallis with Dunn's multiple comparison test. Data from A, B, C and D are representative of one experiment. Data from E and F are pooled data from two independent experiments and were analysed by Kruskal-Wallis with Dunn's multiple comparison test. *P<0.05, **P<0.01, ***P<0.001, ****P<0.0001.

Suppl Fig 1

Table 1: antibodies used for flow cytometry.

antigen	clone	conjugate	Source
CD24	M1/69	FITC	eBioscience (11-0242-82)
Podoplanin	8.1.1	Pe-Cy7	Biolegend (127411)
CD326 (EpCAM)	G8.8	Pe-Dazzle594	Biolegend (118235)
CD31	390	BV605	Biolegend (102427)
MHC-II	M5/114.15.2	AF700	Biolegend (107622)
CD45	30-F11	BV785	Biolegend (103149)
CD140a	APA5	BV421	Biolegend (135923)
Lyve1	ALY7	eFluor660	eBioscience (50-0443-82)
Sca-1	D7	BV711	Biolegend (108131)
Ly6G	1A8	PerCP-Cy5.5	Biolegend (127615)
Gr1	RB6-8C5	PerCP-Cy5.5	Biolegend (108427)
CD4	GK1.5	FITC	Biolegend (100405)
CD90.2	30-H12	PerCP-Cy5.5	Biolegend (105337)
ST2	RMST2-33	APC	eBioscience (17-9333-82)
IL-5	TRFK5	PE	Biolegend (504303)
IL-13	W17010B	PE-Cy7	Biolegend (159407)
SiglecF	E50-2440	BV421	BD Biosciences (565934)
TCRd	GL3	BV421	Biolegend (118119)
Ly6C	HK1.4	BV510	Biolegend (128033)
CD11c	N418	BV605	Biolegend (117333)
CD4	RM4-5	BV650	Biolegend (100555)
CD8	53-6.7	BV711	Biolegend (100747)
CD64	X54-5/7.1	PE	Biolegend (139304)
SiglecF	E50-2440	PE-CF594	BD Biosciences (562757)
CD3e	145-2C11	PE-Cy5	Biolegend (100310)
CD19	6D5	PE-Cy5.5	Biolegend (115510)
Ly6G	1A8	PE-Cy7	Biolegend (127617)
Mertk	2B10C42	APC	Biolegend (151508)
CD11b	M1/70	APC-fire750	Biolegend (101262)
Mouse Hematopoietic Lineage Biotin Panel	Mixed	NA	eBioscience (88-7774-75)
Streptavidin	NA	APC-eFluor780	eBioscience (47-4317-82)

Table 2: antibodies used for immuno-staining.

antigen	antibody clone	dilution	source (catalogue number)
IL-33	rabbit polyclonal	1:1000	Abcam (ab118503)
SPC	rabbit polyclonal	1:1000	Abcam (ab90716)
S100a4	rabbit polyclonal	1:200	Bio Legend (810101)
Scgb1a1	rabbit polyclonal	1:200	Novus Biologicals (NBP1-57961)
Ym1-biotin	goat polyclonal	1:100	R&D Systems (BAF2446)
